# The change of inflammatory markers may predict long-term major adverse cardiovascular events in elderly patients with coronary heart disease: a retrospective cohort study

**DOI:** 10.3389/fmed.2024.1523581

**Published:** 2025-01-13

**Authors:** Li He, Sisi Chen, Xuan Zhu, Fang He

**Affiliations:** Department of Emergency, Wuhan Fourth Hospital, Wuhan, Hubei, China

**Keywords:** monocytes, high-density lipoprotein cholesterol, coronary heart disease, elderly patients, monocyte/HDL

## Abstract

**Background:**

At present, the relationship among inflammatory markers [monocytes/HDL-c (MHR), neutrophils/HDL-c (NHR) and lymphocytes/HDL-c (LHR)] and long-term prognosis of coronary heart disease (CHD) is still unclear. Therefore, this study explores the relationship between inflammatory indicators and the risk of long-term major adverse cardiovascular events (MACE) in elderly patients with CHD.

**Methods:**

A retrospective analysis was conducted on 208 elderly patients who underwent coronary angiography at Wuhan Fourth Hospital from August 2022 to August 2023. They were divided into the CHD group (*N* = 116) and control group (*N* = 92). Patients in the CHD group were followed up for 1 year and divided into the MACE group (*N* = 36) and the non-MACE group (*N* = 80) according to whether MACE occurred.

**Results:**

In elderly patients, logistic regression analysis shows that MHR is an independent risk factor for CHD (OR = 3.050, 95% CI 1.318–1.772). ROC curve analysis found that MHR (AUC = 0.865, 95% CI 0.811–0.919, *p* < 0.001) is higher than NHR and LHR. In patients with CHD, the spearman analysis show that MHR is positively correlated with Gensini score (*R* = 0.266, *p* = 0.004). The logistic regression analysis found that MHR is independent risk factors for MACE (OR = 6.048, 95% CI 1.224–1.941, *p* = 0.002). ROC analysis showed that the critical value of MHR to predict MACE was 0.651, the sensitivity of 58.3% and specificity of 90.0% could predict MACE, and the AUC was 0.793 (95% CI 0.702–0.884, *p* < 0.001) is higher than LHR.

**Conclusion:**

In elderly patients, MHR is an independent predictor of CHD and long-term MACE and is positively correlated with the severity of coronary artery lesions.

## 1 Introduction

As the global population ages, the population aged 80 and over (i.e., the oldest population) is growing even faster than the total number of older people. Between 2008 and 2040, the population over 65 is expected to increase by 160%, while the number over 80 will increase by 233% (1, 2). By 2050, China’s population aged 80 and over is expected to quadruple, accounting for the highest proportion of the oldest age group (3). At the same time, the prevalence and severity of coronary heart disease (CHD) increases significantly with age (4). Elderly patients with CHD are a special group of patients, its pathophysiological mechanism is rather complex, and they are more likely to have multiple other metabolic diseases, which makes the target organ damage of elderly CHD more extensive and leads to a higher incidence rate of major adverse cardiovascular events (MACE) (5). Moreover, the clinical manifestations of elderly CHD patients are often atypical, and the clinical missed diagnosis rate and misdiagnosis rate are as high as 65% (6). At present, in clinical practice, CHD can be accurately and effectively diagnosed through invasive examinations such as intravascular ultrasound (IVUS) and coronary angiography (CAG). These examination techniques are invasive, harmful and costly, and are not easily accepted by elderly patients in clinical settings. If CHD and its prognosis can be predicted and evaluated through some biochemical indicators, it will be of great significance for the timely detection, prediction of CHD, and the anticipation of its severity, treatment effect and prognosis.

Inflammatory reaction and lipid dysregulation is an important part of the pathophysiological mechanism of CHD (7, 8). Neutrophils (NEU), monocytes (MONO) and lymphocytes (LYM) in white blood cells are typical inflammatory cells. Among lipoprotein components, high-density lipoprotein cholesterol (HDL-c) is a protective factor for cardiovascular diseases (CVD) (9). At present, combined markers based on routine peripheral blood cell detection and biochemical detection are attracting more and more attention. NEU to HDL-c (NHR), MONO to HDL-c (MHR), and LYM to HDL-c (LHR) have been recommended as potential markers of systemic inflammation and oxidative stress in many inflammatory diseases (10). Some studies have found that a high ratio of MHR is related to the severity of coronary atherosclerosis (AS) and cardiac events (11). The NHR and LHR are also easily accessible potential inflammatory indicators. They are related to the process of metabolic syndrome and AS formation and can be used as potential indicators of prothrombotic and pro-inflammatory states (12). At present, the relationship between NHR, MHR, LHR and the severity and long-term prognosis of CHD in patients over 80 years is still unclear. Therefore, this study explores the correlation between inflammatory indicators and CHD and the predictive value for the prognosis of elderly CHD patients. By detecting the levels of inflammatory markers, the severity of the disease and cardiovascular risk of elderly CHD patients can be evaluated, providing an important reference for clinical treatment.

## 2 Method

### 2.1 Study design and subjects

A retrospective analysis was conducted on 208 elderly patients who underwent CAG at Wuhan Fourth Hospital from August 2022 to August 2023 were selected, and all patients met any of the following criteria in inclusion and exclusion. Inclusion criteria: (1) CAG was performed during hospitalization; (2) Aged ≥80 years; (3) Had complete relevant clinical and laboratory examination data. Exclusion criteria: (1) Taking drugs that affect blood cells recently; (2) Patients with severe liver and kidney insufficiency; (3) Patients with severe valvular heart disease, severe heart failure, congenital heart disease, acute pulmonary embolism, acute stroke, and respiratory failure; (4) Patients with hematological system diseases, autoimmune diseases, acute and chronic hemorrhagic diseases, and malignant tumors; (5) Data missing, including hematological parameters such as (NEU, MONO, LYM and HDL-c). According to the examination results, they were divided into the CHD group and the non-CHD group (control group). Patients in the CHD group were followed up for 1 year and divided into the MACE group and the non-MACE group according to whether MACE occurred. This study followed the principles of the Declaration of Helsinki, and was approved by the Ethics Committee of Wuhan Fourth Hospital (Ethics Approval No. KY2024-200-01). Informed consent was waived due to the retrospective nature of this study. All personal information regarding patient identity was removed.

### 2.2 Data collection

Retrieve general clinical data from the electronic medical records of all patients, including age, sex, smoking, alcohol consumption, hypertension, T2DM, etc. All patients were fast than 8 h, and fasting venous blood was collected in the morning of the next day. Collect all patient-related laboratory tests, serum TC, TG, HDL-c, low-density lipoprotein (LDL-c), total cholesterol (TG), triglyceride (TC), uric acid (UA), uric acid (UA), glucose (GLu) and other indicators. They were measured using an automated analyzer (Abbott CI8200, Mindray, China). All the test indicators were completed in the laboratory of our hospital. The same instruments and equipment and appropriate reagents were used for testing. The test results were recorded in the same excel spread sheet by specialized researchers.

### 2.3 Definitions

(1) Patients with the coronary involvement was defined as lesion stenosis ≥50% in any epicardial coronary artery with a diameter ≥ 2 mm which were included in the CHD group, while the other patients were included in the control group (13).
(2) Hypertension is defined as systolic blood pressure (SBP) ≥ 140 mmHg and/or diastolic blood pressure (DBP) ≥ 90 mmHg, or current is using antihypertensive drugs (14).
(3) The diagnosis of T2DM is based on one or more of the followings: ① Self-reported history of diabetes, ② Taking hypoglycemic drugs, ③ Fasting blood glucose (FBG) ≥ 7.0 mmol/L, ④ Blood glucose≥11.1 mmol/L 2 h after oral glucose tolerance test (OGTT) was the standard diagnosis, ⑤ HbA1c ≥ 6.5% (15, 16).
(4) CAG and Gensini (GS) score to evaluate the severity of coronary artery stenosis. GS score was used as a quantitative index for the severity of coronary lesions. The severity of coronary artery stenosis was evaluated according to GS score and the score of the degree of stenosis in each coronary artery was the score of the lesion site (a total of 7 coronary artery lesion sites) × the score of the degree of stenosis (6 coronary artery stenosis degrees), and the total score was the sum of all the lesion points. Completed by professional coronary intervention physicians in the cardiac catheterization laboratory of Wuhan Fourth Hospital. All patients choose the arterial route and use multi-position projection to perform CAG to evaluate the severity of coronary artery stenosis (17, 18). According to the coronary artery segmentation evaluation standard of the American Heart Association, the GS scoring system is used to score the severity of coronary artery stenosis.

### 2.4 Clinical outcomes

MACE includes myocardial infarction, heart failure, cardiac arrest, stroke, etc. If rescue is not timely, it can still lead to the death of the patient. At least two attending physicians with clinical experience in cardiovascular and cerebrovascular diseases manually view the electronic medical records of hospitalization or outpatient visits during the follow-up period, and finally confirm whether there is an end event in combination with telephone follow-up (19).

### 2.5 Statistical analysis

SPSS27.0 software was used for statistical analysis. Measurement data conforming to a normal distribution are expressed as mean ± standard deviation and independent sample t-test or ANOVA is used for comparison between groups. Measurement data that do not conform to a normal distribution are expressed as M (P25, P75) and the Mann–Whitney U test is used for comparison between groups. Count data are expressed as cases or cases (%) and the chi-square test is used for comparison between groups. Spearman correlation analysis was used to analyze the correlation between serological markers and GS score of coronary artery lesions; univariate and multivariate logistic regression analysis was used to analyze risk factors and ROC curve was used to judge the accuracy of the model. A difference was considered statistically significant when *p* < 0.05.

## 3 Results

### 3.1 General data analysis

A total of 258 patients underwent CAG. According to the exclusion criteria, 208 patients were included in this study. There were 74 males and 134 females. According to the CHD diagnostic criteria, they were divided into the CHD group (*N* = 116) and the control group (*N* = 92). Compared with the control group, the proportion of hypertension, antilipidemic drug therapy, age, MONO, NEU, NHR, LHR, MHR was significantly increased in CHD group (*p* < 0.05). The levels of HDL-c was significantly decreased in CHD group (*p* < 0.05). However, there were no significant differences in smoking, alcohol consumption, T2DM, sex, LDL-c, TG, TC, UA, GLU and LYM between the CHD group and the control group (Table 1).

**Table 1:** Clinical and laboratory characteristics of patients.

Parameters	Control group (*N* = 92)	CHD group (*N* = 116)	*p*
Sex (*n* %)	33 (35.8)	41 (45.3)	0.937
Age (year)	81 (80,82)	83 (81,84)	<0.001
Smoking [*n* (%)]	9 (9.7)	13 (11.2)	0.740
Alcohol consumption (*n* %)	5 (5.4)	4 (3.4)	0.484
Hypertension [*n* (%)]	59 (64.1)	92 (79.3)	0.015
T2DM [*n* (%)]	20 (21.7)	38 (32.7)	0.078
Antilipidemic drug therapy [*n* (%)]	67 (72.8)	109 (93.9)	<0.001
MONO(×10^9^/L)	0.39 (0.32,0.52)	0.47 (0.37,0.61)	0.005
NEU(×10^9^/L)	2.74 (1.90,4.24)	4.13 (3.18,6.38)	<0.001
LYM(×10^9^/L)	1.41 (1.10,1.69)	1.36 (0.97,1.68)	0.204
HDL-c(mmol/L)	1.17 (1.00,1.43)	1.15 (0.95,1.28)	0.021
LDL-c(mmol/L)	1.98 (1.58,2.72)	2.15 (1.57,2.78)	0.854
TG(mmol/L)	1.08 (0.77,1.60)	1.09 (0.82,1.57)	0.671
TC(mmol/L)	3.82 (3.32,4.51)	3.82 (3.30,4.49)	0.828
UA (μmol/L)	312 (272,399)	350 (266,413)	0.386
GLU(mmol/L)	6.21 (5.44,7.76)	6.21 (5.22,8.41)	0.862
MHR	2.51 (1.71,3.69)	3.74 (2.80,5.65)	<0.001
NHR	2.68 (1.73,4.03)	4.76 (3.74,6.49)	<0.001
LHR	1.13 (0.83,1.57)	1.60 (1.15,2.14)	<0.001

T2DM, Type 2 diabetes; TG, triglycerides; TC, total cholesterol; LDL-c, low-density lipoprotin cholesterol; HDL-c, high-density lipoprotein cholesterol; GLU, glucose; UA, uric acid; NEU, Neutrophil; LYM, lymphocyte; MONO, monocyte; MHR, monocyte high-density lipoprotein cholesterol ratio; LHR, lymphocyte high-density lipoprotein cholesterol ratio; NHR, Neutrophil high-density lipoprotein cholesterol ratio; CHD, coronary heart disease CHD.

### 3.2 Logistic regression analyses of independent predictors of CHD

The dependent variable is whether CHD is diagnosed. Univariate logistic regression analysis is used. The results show that age, hypertension, antilipidemic drug therapy, NEU, MONO, HDL-c, NHR, LHR and MHR are all statistically significant. To avoid multicollinearity, we did not include NEU, LYM, MONO and HDL-c in the regression model. Finally, age, hypertension, NHR, LHR, and MHR were included as independent variables in the multivariate logistic regression analysis. The results show that MHR (OR = 3.050, 95% CI 1.318–1.772, *p* < 0.001) and NHR (OR = 1.233, 95% CI 1.024–1.486, *p* < 0.001) are independent risk factors for the occurrence of CHD (Table 2).

**Table 2:** Logistic regression analyses of independent predictors of CHD.

	Univariate		Multivariate	
Variables	*p*	OR (95% CI)	*p*	OR (95% CI)
Sex	0.937	1.023 (0.578,1.812)		
Age	<0.001	1.304 (1.125,1.512)	0.085	1.165 (0.979–1.386)
Smoking	0.740	1.164 (0.474,2.856)		
Alcohol consumption	0.488	0.621 (0.162,2.383)		
Hypertension	0.016	2.144 (1.154,3.982)	0.112	1.939 (0.857–4.391)
T2DM	0.080	1.754 (0.935,3.290)		
Antilipidemic drug therapy	<0.001	5.810 (2.382,14.172)		
NEU	<0.001	1.538 (1.292,1.832)		
MON	<0.001	9.414 (1.389,1.729)		
LYM	0.369	1.256 (0.764,2.067)		
HDL-C	<0.001	0.006 (0.001,0.032)		
LDL-C	0.799	1.045 (0.743,1.470)		
TG	0.934	1.012 (0.758,1.352)		
TCHO	0.991	0.998 (0.745,1.339)		
UA	0.524	1.001 (0.998,1.004)		
GLU	0.905	0.995 (0.910,1.087)		
MHR	<0.001	5.359 (1.655,1.998)	<0.001	3.050 (1.318–1.772)
NHR	<0.001	1.659 (1.386,1.986)	0.027	1.233 (1.024–1.486)
LHR	<0.001	2.654 (1.637,4.305)	0.636	1.157 (0.632–2.117)

T2DM, Type 2 diabetes; TG, triglycerides; TC, total cholesterol; LDL-c, low-density lipoprotin cholesterol; HDL-c, high-density lipoprotein cholesterol; GLU, glucose; UA, uric acid; NEU, Neutrophil; LYM, lymphocyte; MONO, monocyte; MHR, monocyte high-density lipoprotein cholesterol ratio; LHR, lymphocyte high-density lipoprotein cholesterol ratio; NHR, Neutrophil high-density lipoprotein cholesterol ratio; CHD, coronary heart disease CHD.

### 3.3 ROC curve analysis of MHR, NHR, LHR for predicting CHD

**Figure 1:**
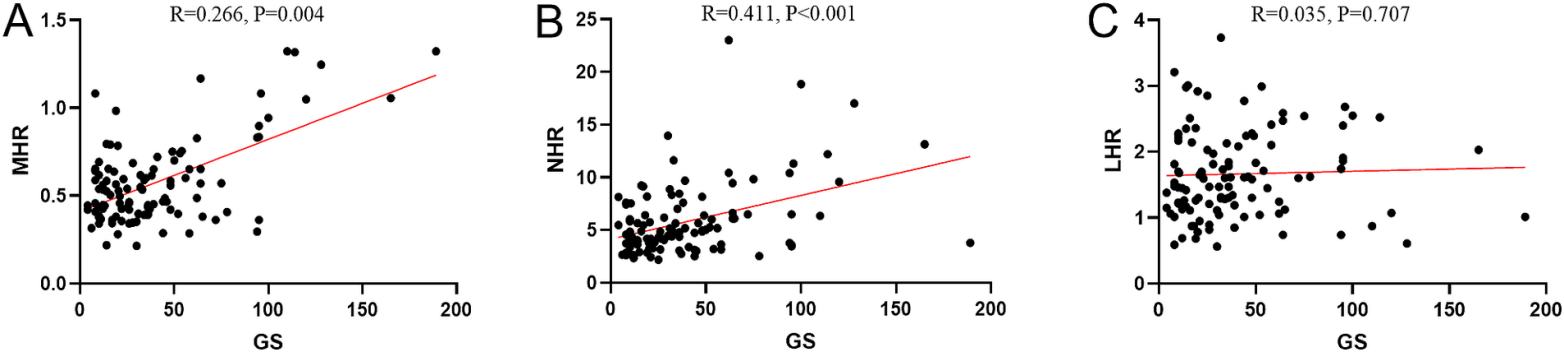
ROC curve analysis of MHR, NHR, LHR for predicting CHD. MHR, monocyte high-density lipoprotein cholesterol ratio; LHR, lymphocyte high-density lipoprotein cholesterol ratio; NHR, Neutrophil high-density lipoprotein cholesterol ratio.

The ROC curve was used to evaluate the predictive effect of NHR, LHR and MHR on the occurrence of CHD. The results show that MHR has certain predictive value for CHD. The area under the curve (AUC) of its index is 0.865 (95% CI 0.811–0.919, *p* < 0.001). The predictive ability of MHR is higher than that of NHR (AUC = 0.802, 95% CI 0.741–0.864, *p* < 0.001) and LHR (AUC = 0.687, 95% CI 0.614–0.760, *p* < 0.001; Figure 1).

### 3.4 Correlation between MHR and GS score

**Figure 2:**
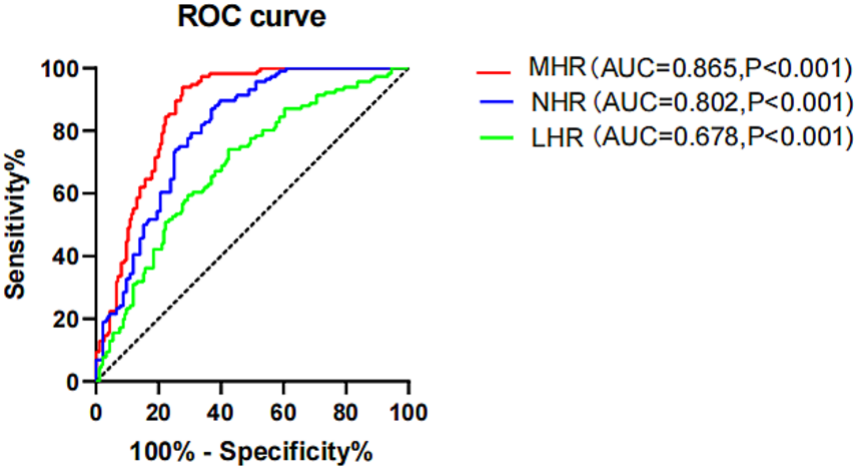
Correlation between MHR, NHR, LHR and Gensini score. MHR, monocyte high-density lipoprotein cholesterol ratio; LHR, Lymphocyte high-density lipoprotein cholesterol ratio; NHR, Neutrophil high-density lipoprotein cholesterol ratio.

The results of spearman correlation analysis show that age (*R* = 0.206, *p* = 0.027), NEU (*R* = 0.373, *p* < 0.001), MHR (*R* = 0.266, *p* = 0.004), and NHR (*R* = 0.411, *p* < 0.001) are positively correlated with GS score. However, sex, smoking, alcohol consumption, hypertension, T2DM, MONO, LYM, HDL-c, LDL-c, TG, TG, UA, GLU and LHR are not correlated with GS score (Table 3; Figure 2).

**Table 3:** The correlation between each factor and Gensini score.

	Gensini score	
	*R*	*p*
Sex (*n* %)	−0.118	0.205
Age (year)	0.206	0.027
Smoking [*n* (%)]	0.004	0.968
Alcohol consumption (*n* %)	−0.110	0.241
Hypertension [*n* (%)]	0.127	0.174
T2DM [*n* (%)]	0.038	0.688
MONO(×10^9^/L)	0.051	0.586
NEU(×10^9^/L)	0.373	<0.001
LYM(×10^9^/L)	−0.046	0.621
HDL-c(mmol/L)	−0.072	0.443
LDL(mmol/L)	0.170	0.069
TG(mmol/L)	0.015	0.877
TC(mmol/L)	0.142	0.127
UA (μmol/L)	0.065	0.490
GLU(mmol/L)	0.141	0.130
MHR	0.266	0.004
NHR	0.411	<0.001
LHR	0.035	0.707

TG, triglycerides; TC, total cholesterol; LDL-c, low-density lipoprotin cholesterol; HDL-c, high-density lipoprotein cholesterol; GLU, glucose; UA, uric acid; NEU, Neutrophil; LYM, lymphocyte; MONO, monocyte; MHR, monocyte high-density lipoprotein cholesterol ratio; LHR, lymphocyte high-density lipoprotein cholesterol ratio; NHR, Neutrophil high-density lipoprotein cholesterol ratio.

### 3.5 Predictive value of inflammatory indicators for MACE occurrence in CHD patients after 1 year

One hundred and sixteen CHD patients were followed up and divided into the MACE group (*N* = 36) and the non-MACE group (*N* = 80) according to whether MACE occurred. Older age and the proportion of hypertensive were determined at significantly higher rates in MACE group than in non-MACE group (*p* = 0.004, *p* = 0.028). The MONO, LYM, LHR and MHR values were determined to be significantly higher (*p* < 0.001, *p* = 0.008, *p* < 0.001, *p* = 0.003, respectively) in MACE group than in non-MACE group. The median values of the MHR index for the two groups of patients were 0.45 (0.39,0.59) and 0.71 (0.54,0.88). There was no statistically significant difference in proportion of sex, T2DM, antilipidemic drug therapy, smoking, alcohol consumption and HDL-c, LDL-c, TG, TG, UA, GLU between the two groups (*p* > 0.05; Table 4).

**Table 4:** Comparison of clinical data of CHD patients between non-MACE group and MACE group.

Parameters	Non-MACE group (*N* = 80)	MACE group (*N* = 36)	*p*
Sex (*n* %)	26 (32.5)	15 (41.6)	0.339
Age (year)	82 (80,84)	83 (82,85)	0.004
Smoking [*n* (%)]	6 (7.5)	7 (19.4)	0.059
Alcohol consumption (*n* %)	2 (2.5)	2 (5.5)	0.404
Hypertension [*n* (%)]	59 (73.7)	33 (91.6)	0.028
T2DM [*n* (%)]	26 (32.5)	12 (33.2)	0.930
Antilipidemic drug therapy [*n* (%)]	74 (92.5)	35 (97.2)	0.323
MONO(×10^9^/L)	0.41 (0.33,0.50)	0.59 (0.49,0.74)	<0.001
NEU(×10^9^/L)	4.08 (3.09,6.31)	4.44 (3.55,7.11)	0.193
LYM(×10^9^/L)	1.34 (1.04,1.60)	1.62 (1.22,1.92)	0.008
HDL-c(mmol/L)	0.90 (0.76,1.04)	0.89 (0.73,0.98)	0.400
LDL-c(mmol/L)	2.02 (1.45,2.77)	2.24 (1.98,2.77)	0.117
TG(mmol/L)	1.11 (0.83,1.49)	1.08 (0.82,1.64)	0.481
TC(mmol/L)	3.74 (3.28,4.49)	3.92 (3.42,4.45)	0.363
UA (μmol/L)	348 (270,413)	352 (255,416)	0.978
GLU(mmol/L)	6.22 (5.21,9.13)	6.21 (5.49,7.37)	0.962
MHR	0.45 (0.39,0.59)	0.71 (0.54,0.88)	<0.001
NHR	4.71 (3.65,6.29)	5.35 (3.78,8.96)	0.164
LHR	1.47 (1.13,1.80)	1.97 (1.29,2.45)	0.003

T2DM, Type 2 diabetes; TG, triglycerides; TC, total cholesterol; LDL-c, low-density lipoprotin cholesterol; HDL-c, high-density lipoprotein cholesterol; GLU, glucose; UA, uric acid; NEU, Neutrophil; LYM, lymphocyte; MONO, monocyte; MHR, monocyte high-density lipoprotein cholesterol ratio; LHR, lymphocyte high-density lipoprotein cholesterol ratio; NHR, Neutrophil high-density lipoprotein cholesterol ratio; CHD, coronary heart disease CHD.

Univariate logistic regression analysis shows that age, hypertension, MHR, MONO, NEU, NHR, and NHR are statistically significant. Including age, hypertension, MHR, NHR, and LHR in the multivariate logistic regression model found that MHR and age are independent risk factors for MACE occurrence in CHD patients after 1 year (OR = 6.048, 95% CI 1.224–1.941, *p* = 0.002; Table 5).

**Table 5:** Logistic regression analyses of predictors of long-term MACE in CHD patients.

	Univariate		Multivariate	
Variables	*p*	OR (95% CI)	*p*	OR (95% CI)
Sex	0.341	0.674 (0.300,1.517)		
Age (year)	0.004	1.293 (1.086,1.539)	0.008	1.314 (1.074–1.608)
Smoking	0.068	2.977 (0.922,9.610)		
Alcohol consumption	0.416	2.294 (0.310,16.967)		
Hypertension	0.037	3.915 (1.086,14.118)	0.097	3.945 (0.779–19.97)
T2DM	0.930	1.038 (0.450,2.396)		
Antilipidemic drug therapy	0.343	2.838 (0.329,24.481)		
NEU	0.146	1.137 (0.956,1.353)		
MON	0.001	64.257 (5.791,713.022)		
LYM	0.033	2.289 (1.071,4.892)		
HDL-C	0.250	0.263 (0.027,2.557)		
LDL-C	0.364	1.245 (0.776,1.999)		
TG	0.066	1.454 (0.976,2.166)		
TCHO	0.484	1.154 (0.773,1.722)		
UA	0.572	1.001 (0.997,1.005)		
GLU	0.949	0.995 (0.865,1.146)		
MHR	<0.001	1.245 (1.103,1.653)	0.002	6.048 (1.224–1.941)
NHR	0.042	1.131 (1.005,1.274)	0.613	1.038 (0.898–1.201)
LHR	0.005	2.451 (1.302,4.615)	0.078	1.968 (0.926–4.184)

T2DM, Type 2 diabetes; TG, triglycerides; TC, total cholesterol; LDL-c, low-density lipoprotin cholesterol; HDL-c, high-density lipoprotein cholesterol; GLU, glucose; UA, uric acid; NEU, Neutrophil; LYM, lymphocyte; MONO, monocyte; MHR, monocyte high-density lipoprotein cholesterol ratio; LHR, lymphocyte high-density lipoprotein cholesterol ratio; NHR, Neutrophil high-density lipoprotein cholesterol ratio; CHD, coronary heart disease CHD; MACE, major adverse cardiovascular event.

**Figure 3:**
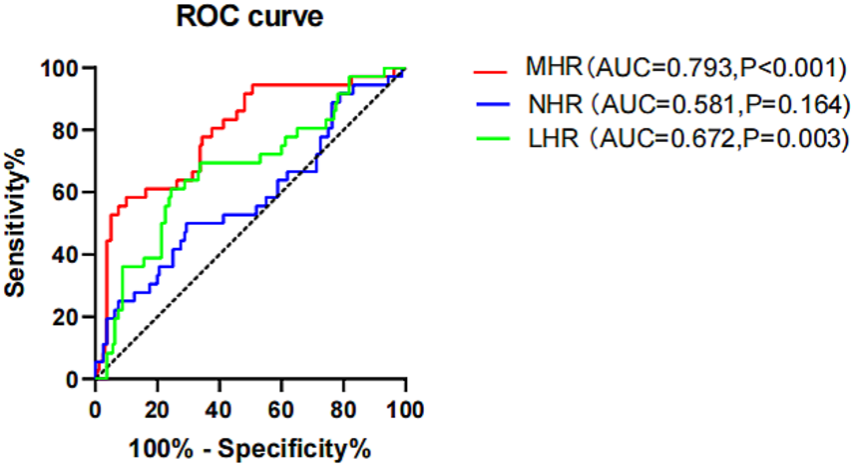
ROC curve analysis of MHR, NHR and LHR for predicting long-term MACEs in CHD patients. MHR, monocyte high-density lipoprotein cholesterol ratio; LHR, lymphocyte high-density lipoprotein cholesterol ratio; NHR, Neutrophil high-density lipoprotein cholesterol ratio.

In order to further study and compare the prediction effect of MHR, NHR and LHR, we draw the ROC curve. Of the three indicators examined, MHR had the highest AUC of 0.793 (95% CI 0.702–0.884, *p* < 0.001). The optimal cut-off value of MHR is 0.651. The sensitivity for predicting MACE is 58.3% and the specificity is 90.0%. LHR was next with an AUC of 0.672 (95% CI 0.562–0.781, *p* = 0.003) with an optimal threshold of 1.795 and an optimal cut-off value 5.649 (sensitivity 61.1%, specificity 75.0%). However, the lowest AUC was found for NHR with an AUC of 0.581(95% CI: 0.464–0.698, *p* = 0.164) with no statistically significant (Figure 3). In short, this means that when MHR is higher than 0.651, we need to pay extra attention because they are high-risk groups for MACE. Clinicians need to urge them to improve their lifestyle, promote healthy diet and physical activities to help them prevent or delay MACE early.

## 4 Discussion

The occurrence and development of CHD are affected by multiple factors. Risk factors that have been identified, such as smoking, T2DM, hyperlipidemia, hypertension, type A personality, obesity and genetic factors (20). Further understanding of its molecular pathogenesis and doing a good job in primary prevention are of crucial importance. It has been clearly established that AS is an independent risk factor for future adverse cardiovascular events. It is the deposition of lipid substances on the vessel wall. In recent years, more and more studies have shown that biomarkers from peripheral blood, such as MHR, LHR and NHR, can replace conventional markers of inflammatory status (IL-6, CRP, and adiponectin) (21, 22). They can be used as indicators to detect and predict the presence and severity of systemic inflammatory processes, including CVD (23). Therefore, in this study, we explored the relationship between inflammatory indicators (MHR, LHR, NHR) and CHD in elderly patients, and proposed that MHR can better predict the risk of long-term MACE in elderly CHD patients. The results found that MHR has value in predicting the occurrence of adverse cardiovascular events in elderly CHD patients. The highest AUC of MHR is 0.793 (95% CI 0.702–0.884, *p* < 0.001). Correlation analysis also shows that MHR is positively correlated with GS score, indicating that the higher the MHR, the more severe the CHD. The results of logistic regression analysis show that MHR is an independent risk factor for the occurrence of CHD (OR = 3.050, 95% CI 1.318–1.772, p < 0.001) and long-term MACE in CHD patients (OR = 6.048, 95% CI 1.224–1.941, *p* = 0.002). In conclusion, MHR is a new type of marker that can be used to predict the severity of CHD and MACE in elderly CHD patients.

CHD is age-dependent (incidence and prevalence), and for the disorders, aging independently increases risk for adverse outcomes. The prevalence of patients with the combination of advanced age (including the very old, aged ≥80 years) and CHD is increasing (24). Adults ≥75 years of age constitute approximately 30–40% of all hospitalized acute coronary syndrome (ACS) patients and the majority of ACS-related mortality is observed in this segment of the population (25–27). The progressive increase in the incidence of adverse outcomes with increasing age culminated in the very old patients having the highest incidence of each adverse outcome, with the exception of revascularization (24). Therefore, the participants in this study were exclusively individuals aged 80 years and older. Our study also revealed that spearman correlation analysis showed a positive correlation between age and the GS score (*R* = 0.206, *p* = 0.027) and age is an independent predict the occurrence of MACE in patients with CHD (OR = 1.314, 95% CI 1.074–1.608, *p* = 0.008).

Previous studies have shown that inflammatory reactions play an important role in the progression and instability of AS and CVD (28). In patients with active inflammation, an increase in the concentration of inflammatory mediators or markers can be used to predict the occurrence of AS CVD. In the process of AS plaque formation, first, through the interaction of NEU, MONO and endothelial cells, it can affect the uptake and metabolism of lipids. LYM, NEU and MONO release pro-inflammatory cytokines and inflammatory mediators through direct and indirect actions. Inflammatory factors such as interleukin-1, interleukin-6 (IL-6) and tumor necrosis factor-*α* (TNF-α) can stimulate vascular endothelial cells to further express adhesion molecules, increasing the adhesion and recruitment of inflammatory cells (29, 30). Meanwhile, they can also activate vascular smooth muscle cells, prompting their proliferation and migration. And the inflammatory mediators promote the activation and aggregation of platelets, accelerating thrombosis (31). Once a thrombus forms and blocks the coronary artery, it will lead to myocardial ischemia and hypoxia, triggering serious CHD events like acute myocardial infarction (32). At the same time, they will cause apoptosis of vascular smooth muscle cells, reducing the supporting effect on plaques. When plaques are affected by hemodynamic factors (such as blood pressure fluctuations, vasoconstriction, etc.), they are more likely to rupture (33). LYM recognize autoantigens such as ox-LDL through autoimmune reactions to form immune complexes, which further activate the complement system and generate anaphylatoxins, attracting more inflammatory cells to gather on the blood vessel wall and accelerating the formation and development of atherosclerotic plaques (34). NEU contain a rich enzyme system and can produce a large amount of reactive oxygen species (ROS). ROS has a strong oxidizing effect and can oxidize low-density lipoprotein (LDL) to turn it into oxidized LDL (ox-LDL). Ox-LDL is taken up by macrophages to form foam cells, promoting the formation and development of plaques (35, 36). After the vascular endothelium is damaged, chemokines attract MONO to migrate to the subintima, differentiate into macrophages and take up ox-LDL to form foam cells, constituting the lipid core of the plaque. The inflammatory mediators secreted by them prompt the migration of smooth muscle cells, affect the stability of the fibrous cap, and also participate in immunoregulation and vascular remodeling (37, 38). After the plaque ruptures, it can trigger thrombosis that leads to coronary artery obstruction and induces serious consequences such as myocardial infarction (33, 39). However, HDL-c particles exhibit anti-AS properties by removing cholesterol from macrophages, showing antioxidant effects, preventing thrombosis, and maintaining endothelial function and low blood viscosity through the deformability of red blood cells (40). High-level inflammatory markers and low-level HDL-c jointly promote atherosclerosis and contribute to the occurrence of CHD.

The NHR is calculated from NEU and HDL-c. Studies have confirmed that NHR and LHR are not only related to the occurrence and severity of CHD, but also can predict the severity of CHD better than NEU, HDL-c or LDL-c/HDL-c (12, 41). The results of our study found that in elderly patients, LHR is not related to the severity of coronary stenosis, while NHR can effectively predict the occurrence of CHD and is related to the severity of coronary stenosis(*R* = 0.411, *p* < 0.001). MHR is a newly developed inflammatory marker calculated by the ratio of MONO to HDL-c levels. Some scholars have found that MHR may be better than MONO or HDL-c levels alone in predicting the occurrence and progression of AS, thereby predicting coronary events (11). Kanbay et al.’s research shows that a higher MHR is an independent predictor of major cardiovascular events during follow-up in patients with CKD (42). In addition, Kundi et al.’s research shows that MHR is independently and significantly associated with the SYNTAX score in patients with stable CHD (43). This is consistent with our research results. MHR is positively correlated with the severity of coronary arteries (*R* = 0.266, *p* = 0.004); multivariate logistic regression analysis found that MHR is an independent risk factor for the occurrence of CHD and long-term MACE in CHD patients. Additionally, the ROC analysis showed a significant improvement in the ability to identify MACE using the MHR (AUC = 0.793). Therefore, compared with a single cellular component or biochemical index, the predictive value of combined indexes such as MHR, NHR, and LHR may be higher. The effect of MHR is the most optimal.

In summary, in elderly patients, MHR is an independent predictor of CHD and its long-term MACE and is positively correlated with the severity of coronary artery lesions. This predictive ability helps to screen out high-risk groups for CHD in clinical practice so that interventions can be carried out in advance. For patients who have already been diagnosed with CHD, the MHR can be used to monitor changes in their condition. A relatively high MHR is often associated with a poorer prognosis for patients with CHD. A continuously rising ratio may indicate that patients are more likely to experience adverse cardiovascular events, such as recurrent myocardial infarction and heart failure. In our study, during the long-term follow-up of patients with CHD, it has been found that the mortality rate of those patients with a consistently high ratio is significantly higher than that of patients with a normal ratio. This provides a useful reference indicator for clinicians to assess the prognosis of patients.

### 4.1 Strengths and limitations

This is the first study to confirm that MHR has predictive value in elderly CHD patients and is related to the long-term prognosis of CHD patients. These findings provide valuable significance for the screening of elderly CHD and provide an important reference for future research. However, our study has some limitations. First, this is a single-center retrospective study. Data may have missing values due to incomplete records. Retrospective studies are usually observational studies, and it is very difficult to strictly control confounding factors as in experimental studies. However, to increase the reliability of the results, we excluded the missing data in the main research results. Second, this study was conducted in patients who received coronary CAG and cannot represent the general population. There may be selection bias, leading to overestimation or underestimation of the correlation of observed outcomes. Finally, The sample size was relatively small. In the future, we hope that more large-sample, multi-center, prospective trials will prove the clinical role of this indicator and establish a standard range, we anticipate obtaining more favorable outcomes in future research.

## 5 Conclusion

In summary, in elderly patients, MHR is an independent predictor of CHD and its long-term MACE and is positively correlated with the severity of coronary artery lesions. Therefore, MHR has a high predictive value for MACE and severe coronary stenosis in CHD patients, and can provide clinicians with more accurate prognostic information, facilitating timely adoption of appropriate intervention measures to prevent the occurrence of MACE and severe coronary stenosis and improve the overall prognosis of CHD patients.

## Data Availability

The original contributions presented in the study are included in the article/Supplementary material, further inquiries can be directed to the corresponding author/s.

## Glossary

CHD: Coronary heart disease
CAG: Conventional coronary angiography
ROC: Receiver-operating characteristic
GS: Gensini score
CIs: Confidence intervals
AS: Arteriosclerosis
MACE: Major adverse cardiovascular events
CVD: Cardiovascular diseases
SBP: Systolic blood pressure
DBP: Diastolic blood pressure
GS: Gensini
TG: Triglycerides
TC: Total cholesterol
LDL-c: Low-density lipoprotin cholesterol
HDL-c: High-density lipoprotein cholesterol
GLU: Glucose
UA: Uric acid
NEU: Neutrophil
LYM: Lymphocyte
MONO: Monocyte
MHR: Monocyte high-density lipoprotein cholesterol ratio
LHR: Lymphocyte high-density lipoprotein cholesterol ratio
NHR: Neutrophil high-density lipoprotein cholesterol ratio
